# Supplementary material for: Function and clinical relevance of RHAMM isoforms in pancreatic tumor progression
Source: Mol Cancer. 2019 May 9;18:92. doi: 10.1186/s12943-019-1018-y (PMC6506944; doi:10.1186/s12943-019-1018-y)
Supplement: Supplementary file 1 — Figure S1. RT-qPCR analysis of RHAMMA (A) and RHAMMB (B) in 9 primary human PNETs and 3 metastatic PNETs from the livers. Figure S2. Unlike RHAMMB, RHAMMA did not promote liver metastasis of mouse PNET N134 cell line in a tail vein experimental metastasis assay. (A) The number of liver macrometastases was recorded. (B) Immunohistochemical staining of synaptophysin in the liver sections to reveal the presence of metastatic PNETs. Arrows indicate micrometastases. Original magnification: 10X. Scale bar, 200 μm. Figure S3. Correlation between EGFR expression and RHAMMA (A) or RHAMMB (B) expression in TCGA PDAC dataset (RNA-Seq V2). (PPTX 3371 kb) [file 12943_2019_1018_MOESM1_ESM.pptx]

## Slide 1
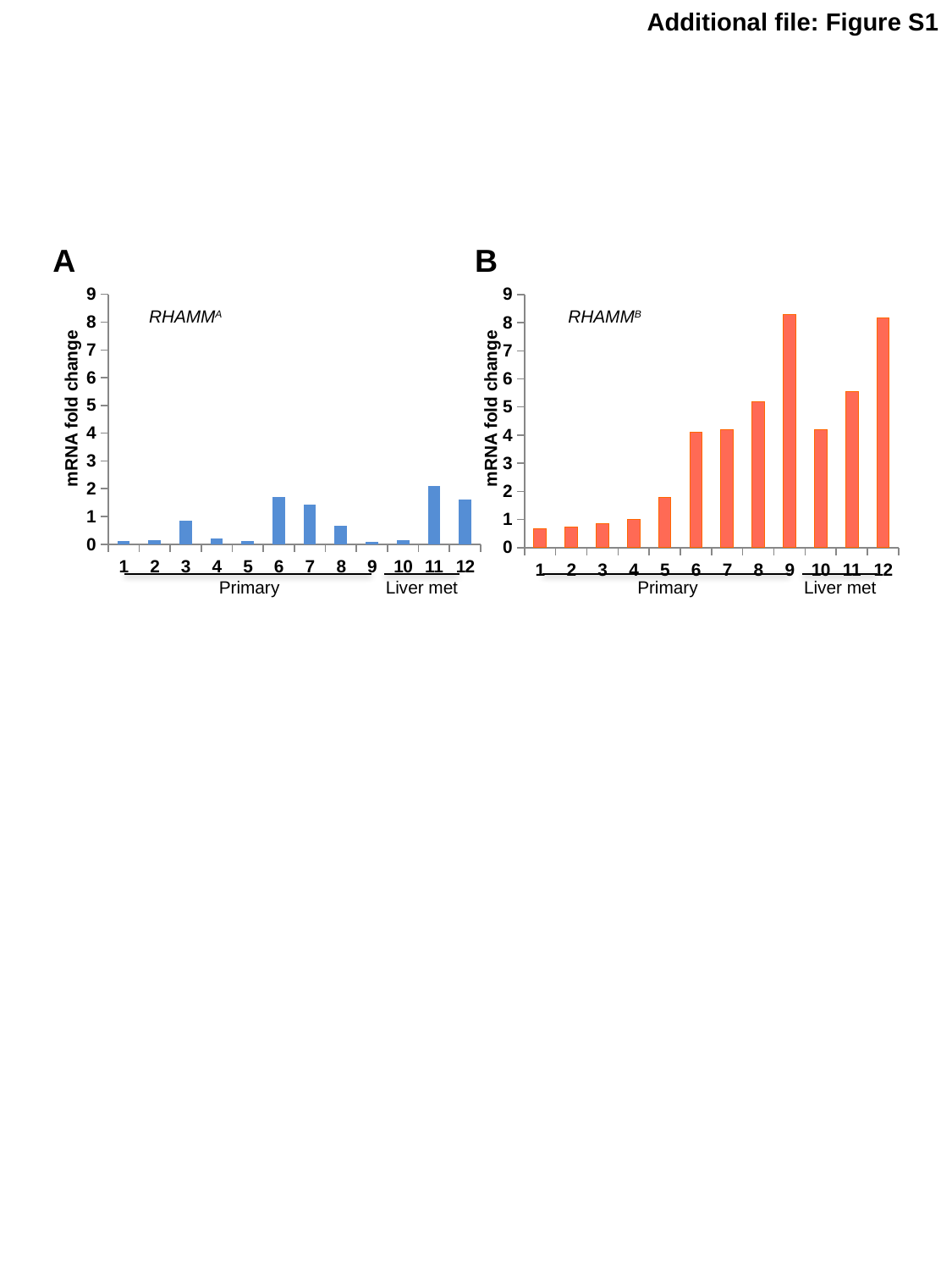

Additional file: Figure S1
A
B
### Chart
| Category |
|---|
### Chart
| Category | |
|---|---|
### Chart
| Category | |
|---|---|RHAMMA
RHAMMB
mRNA fold change
mRNA fold change
 Primary Liver met
 Primary Liver met

## Slide 2
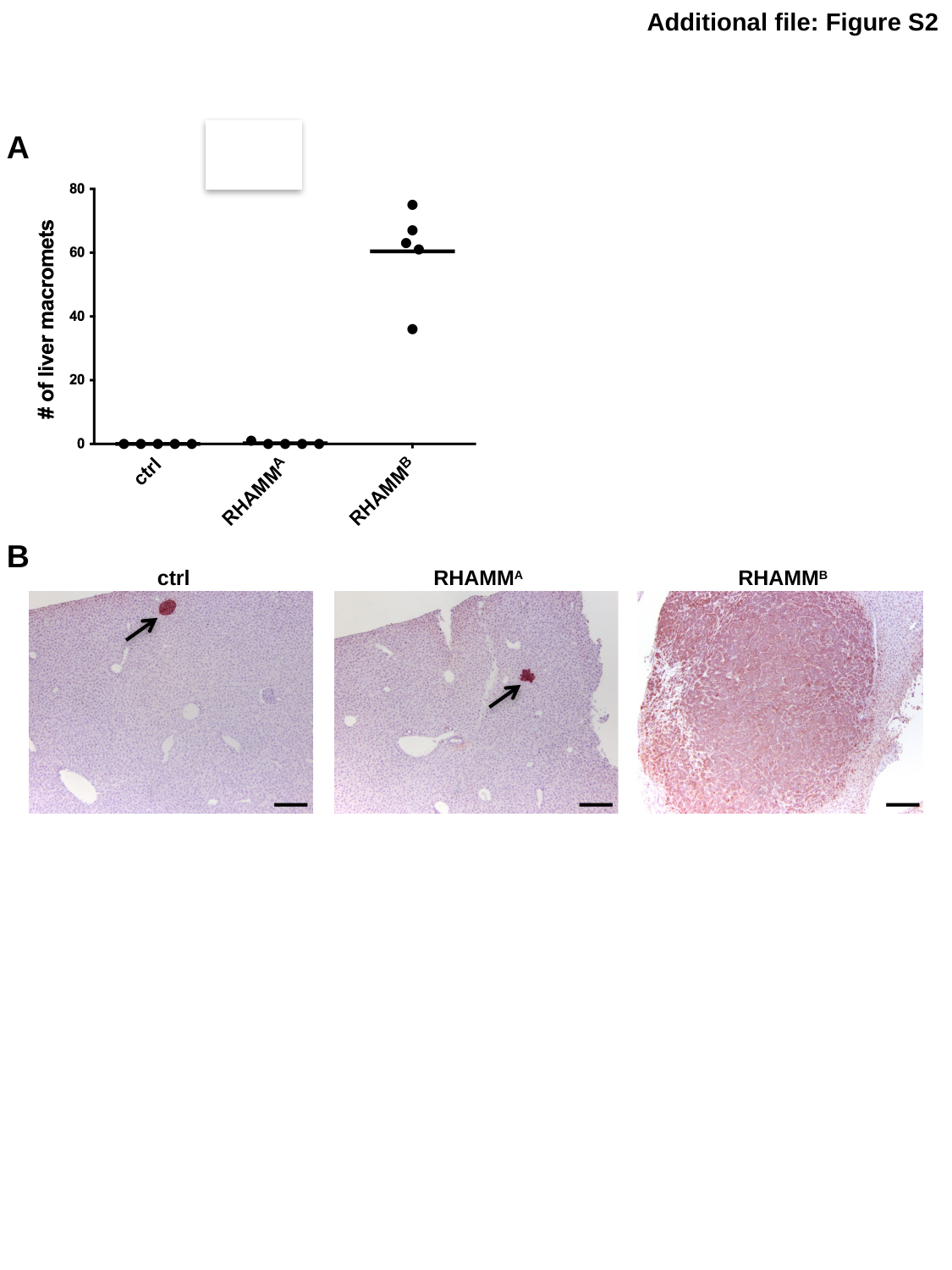

Additional file: Figure S2
C
A
B
ctrl
RHAMMA
RHAMMB

## Slide 3
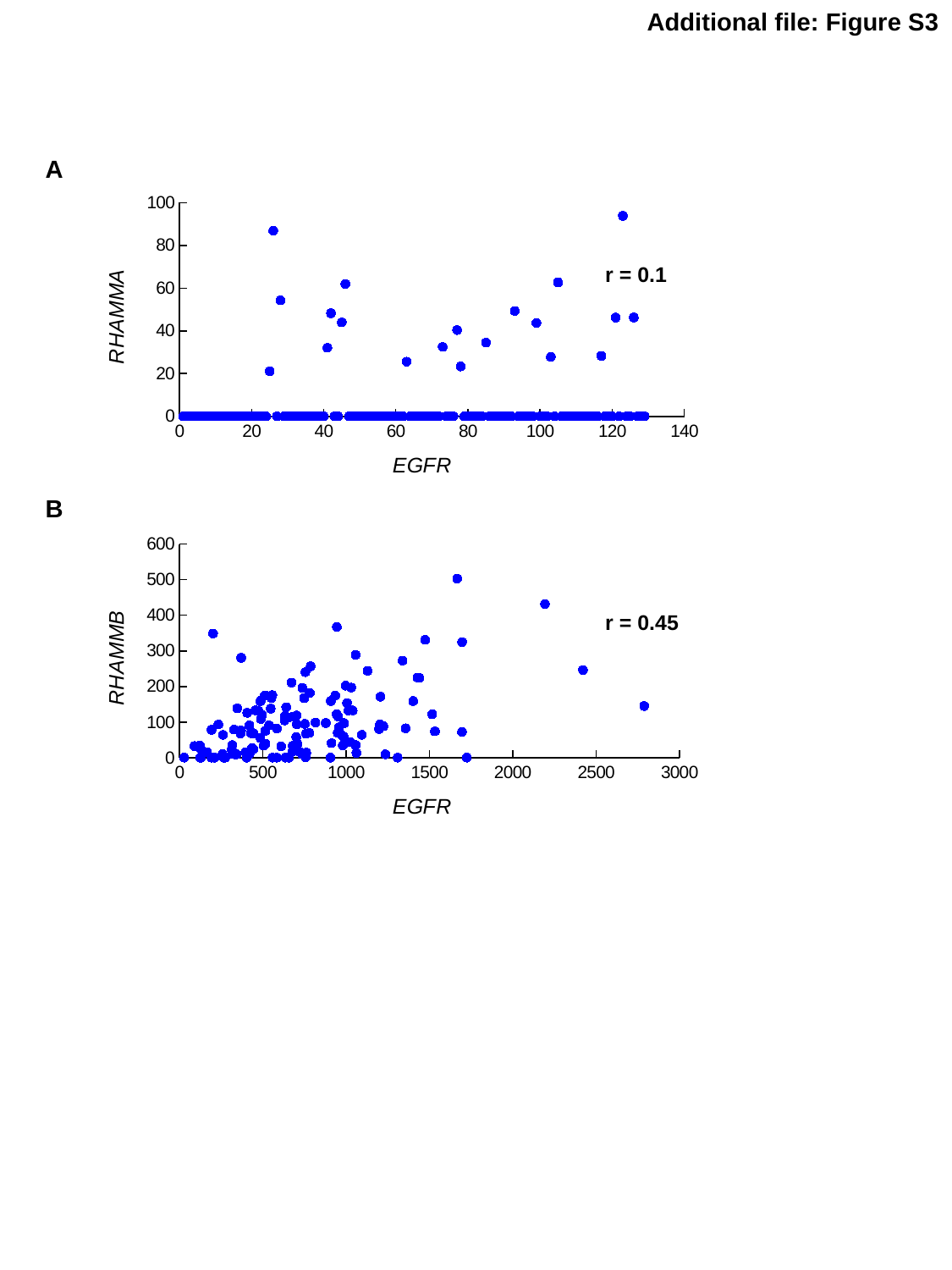

Additional file: Figure S3
A
### Chart
| Category | |
|---|---|r = 0.1
B
### Chart
| Category | RHAMM BETA |
|---|---|r = 0.45
