# Supplementary material for: Function and clinical relevance of RHAMM isoforms in pancreatic tumor progression
Source: Mol Cancer. 2019 May 9;18:92. doi: 10.1186/s12943-019-1018-y (PMC6506944; doi:10.1186/s12943-019-1018-y)
Supplement: Supplementary file 2 — Table S1. Impact of genes on lymph node and liver metastasis of PNETs in RIP-Tag; RIP-tva mice. (DOCX 37 kb) [file 12943_2019_1018_MOESM2_ESM.docx]

**Table S1.** Impact of genes on lymph node and liver metastasis of PNETs in *RIP-Tag; RIP-tva* mice.

| **RCASBP-** | **Age (week)** | **Lymph node metastasis** | **Liver metastasis** |
| --- | --- | --- | --- |
| *RHAMM^A^* | 16 | 0/8 mice (0%) | 0/8 mice (0%) |
| *RHAMM^B^* | 16 | 8/11 mice (73%) | 8/11 mice (73%) |
| *EGFR** | 16 | 5/8 mice (62.5%) | 2/8 mice (25%) |
