# Supplementary material for: Function and clinical relevance of RHAMM isoforms in pancreatic tumor progression
Source: Mol Cancer. 2019 May 9;18:92. doi: 10.1186/s12943-019-1018-y (PMC6506944; doi:10.1186/s12943-019-1018-y)
Supplement: Supplementary file 3 — Supplementary materials and methods. (DOCX 37 kb) [file 12943_2019_1018_MOESM3_ESM.docx]

**Methods and Materials**

**Bioinformatics and Statistical Analysis for *RHAMM* variants from RNA-Seq data**

Total RNAs from 27 primary human PanNETs, 12 metastatic human PanNETs, and human BON1 cell line [1] were isolated with RNeasy Plus Universal Kits (Qiagen Cat no. 73404, Germantown, MD) according to the manufacturer’s protocol. Library preparation and RNA sequencing with paired-end 75 bp reads were performed according to protocols at the Genomics Resources Core Facility, Memorial Sloan Kettering Cancer Center, and Weill Cornell Medicine. A RNA-Seq dataset of 89 human pancreatic islets was obtained from publicly available database Gene Expression Omnibus (GEO accession GSE50398, <http://www.ncbi.nlm.nih.gov/geo/query/acc.cgi?acc=GSE50398>) [2-4], and was converted to fastq file from SRA format using SRA Toolkit (v 2.4.4). All samples analyzed were aligned to hg19 reference genome using STAR (v 2.4) aligner. Aligned samples were then quantified to obtain gene expression in terms of FPKM (Fragments per Kilobase of Transcripts per Million Reads) against a UCSC hg19 annotation Gene Transfer Format containing coordinates for all genes, including specific RHAMM isoforms of interest using CuffLinks (v 2.2.1). FPKM values were extracted from isoform specific quantification output obtained from CuffLinks for each sample, with focus on isoform A (NM_012484) and isoform B of *RHAMM* (NM_012485). Log-transformed FPKM values (log2 [FPKM+1]) were used for further analysis to compare the expression levels across tissue types (Normal, Primary and Liver metastasis) and *RHAMM* isoforms (*RHAMM^A^* and *RHAMM^B^*) using two-way ANOVA, followed by pairwise comparisons with Tukey’s post-hoc test for multiple comparison adjustment. All analyses were performed in open-source data analysis software R (v 2.14.1) or SAS 9.4 (SAS Institute, Cary, NC).

Bioinformatics and statistical analyses were conducted on the publically available gene expression dataset from The Cancer Genome Atlas (TCGA; <http://cancergenome.nih.gov/>). TCGA normalized expression values were created by Illumina RNA-Seq version 2 RNA sequencing data (level 3). Downloaded data was analyzed using R or statistical software Prism (version 6.0f) for statistical computation and Kaplan-Meier survival analysis with log-rank test. Two-tailed Mann-Whitney U test was used to compare differences between two groups selected. P values < 0.05 were considered as statistically significant.

**Quantitative real-time reverse transcription PCR (RT-qPCR)**

Messenger RNA (mRNA) was isolated from human PanNET specimens or cells grown on 6-cm or 10-cm plates using RNeasy mini kit (Qiagen) containing gDNA eliminator spin columns. The cDNA was generated using SuperScript III First-strand synthesis system with random hexamers (Invitrogen), and power SYBR green (Invitrogen)-based qPCR was performed with 3 internal control genes and the comparative C_T_ method (ΔΔC_T_).

The sequences of the primers used are *RHAMM^A^* (forward: located within exon 3, 5’- tgacaaagatactacct tgcctgct-3’, reverse: located at the junction of exon 3 and 4, 5’-tcattcttttgagattcctttgattc-3’); *RHAMM^B^* (forward: located at the junction of exon 3 and 5, 5’-AAAGTTAAGTCTTCG GAATCAAAGATT-3’, reverse: located within exon 5, 5’- GCATTATTTGCA GAGAGAGATGT-3’), and internal control genes: human *HMBS* (forward: 5’-CCATCATCCT GGCAACAGCT-3’, reverse: 5’-GCATTCCTCAGGGTGCAGG-3’); human *EEF1A* (forward: 5’-CAATGTGGGCTTCAA TGTCAA-3’, reverse: 5’-CATAGCCGGCGCTTATTTG-3’); human *MRPL19* (forward: 5’-GGGATTTGCATTCAGAGATCAGG-3’, reverse: 5’-CTCCTGGACCCGAGGATTATAA-3’).

**Tissue preparation, immunohistochemistry, and scoring of protein expression**

Retrospective and prospective review of PanNETs was performed using the pathology files and pancreatic cancer database at the authors’ institutions with Institutional Review Board (IRB) approval. Construction of TMA of human PanNETs was described previously [5]. Mouse tissues were fixed in 10% buffered formalin overnight at room temperature. Fixed tissues were processed and cut into 5 μm sections at Histoserv. Formalin-fixed/paraffin-embedded sections were deparaffinized and rehydrated by passage through a graded xylene/ethanol series before staining. Immunochemistry was examined by VECTASTAIN Elite ABC Kits (Vector Laboratories, Inc., Burlinggame, CA) following manufacturer’s instructions. The primary antibodies used were rabbit anti-RHAMM (1:1,000, Y800 [6], 1:100), rabbit anti-synaptophysin (1:100, Vector Laboratories, VP-S284 or 1:100, Lab Vision/neomarkers, Fremont, CA, RM-9111), and GFP (1:300, Invitrogen, Carlsbad, CA, A11122). RHAMM expression for each tumor was given a score of 0 if no staining was present, and a score of 1 if moderate to strong staining was present.

**Cloning of RCASBP and retroviral vectors**

RCASBP is a replication-competent avian leucosis virus with a splice acceptor and the Bryan-RSV pol gene. RCASBP-*RHAMM^B^* [6] and RCASBP-*EGFR** [7] have been described. RCASBP-*RHAMM^A^* was generated using QuikChange Lightning Site-Directed Mutagenesis Kit (Agilent Technology, Santa Clara, CA) to add exon 4 (forward primer: 5'-agttaagtcttcggaatcaaaggaatctcaaaagaatgataaa gatttgaagatattagagaaagagattcgtgttcttctacaggaac-3', reverse primer: 5'-gttcctgtagaagaacacgaatctctttctctaatatcttcaaatctttatcattcttttgagattcctttgattccgaa

gacttaact-3') from RCASBP-*RHAMM^B^*. The presence of exon 4 in RCASBP-*RHAMM^A^* was confirmed by DNA sequencing.

**The shRNA knockdown**

Hairpin sequences targeting *EGFR* are S7: CCAAGCCAAATGGCATATTTA and S9: GCTTTCGAGAACCTAGAAATA. Hairpin sequence targeting *LacZ* is TCGTATTACAACGTCGTGACT. Hairpin sequence targeting *RHAMM* is sh1: GCCAACTCAAATCGGAAGTAT (Sigma, Clone ID: NM_012484.2-2128s21c1).

Lentiviruses harboring shRNA were generated using 293T cells as described previously (

http://www.broadinstitute.org/rnai/public/resources/protocols).

**Cell culture and Western blot**

Generation of N134 and BON1-TGL cell lines has been described [8, 9]. Human pancreatic neuroendocrine tumor cell line, BON1*,* was provided by Chris Harris [1, 5]. DF1, N134, and BON1-TGL were cultured in Dulbecco’s modified Eagle’s medium (DMEM) supplemented with 10% fetal bovine serum (FBS), 6 mM L-glutamine, and penicillin/streptomycin. The BON1-TGL cell line was transfected with pBABE-*RHAMM^B^* with lipofectamine 2000 (Invitrogen) to generate BON1-TGL-RHAMM^B^ cell line. BON1-TGL cells overexpressing a control vector or *RHAMM^B^* were cultured in DMEM supplemented with 0.5 μg/ml puromycin, 10% FBS, 6 mM L-glutamine, and penicillin/streptomycin. N134 cells overexpressing cDNAs were generated as described [10]. N134 cells overexpressing shRNAand BON1-TGL overexpressing shRNA were cultured in medium supplemented with 0.4 μg/ml puromycin and 0.5 μg/ml puromycin, respectively.

For Western blot analysis, cell extracts were loaded into mini-protean 4-15% pre-cast gels (Bio-Rad). Protein was immobilized onto nitrocellulose membrane, 0.45 µm pore size (Bio-Rad). Blots were blocked for one hour with 3% (weight/volume) bovine serum albumin (Fisher Scientific) and incubated overnight at 4° C with a rabbit monoclonal antibody to RHAMM [EPR4055] antibody at 1:1,000 dilution (Abcam, ab108339). The next day blots were washed 4 times for 10 minutes with TBST and incubated for one hour at room temperature with an anti-rabbit secondary antibody at 1:5,000 dilution. Bands were detected with enhanced chemiluminescence.

**Animal experiments**

Generation of *RIP-Tag; RIP-tva* mice has been described [8] and detailed protocols for somatic delivery of the RCASBP viruses has been described [10]. Immunodeficient mice, NOD/scid-IL2Rgc knockout (NSG), were generated by the Jackson Laboratory. This study was carried out in strict accordance with the recommendations in the Guide for the Care and Use of Laboratory Animals of the National Institutes of Health. All mice were housed in accordance with institutional guidelines. All procedures involving mice were approved by the Institutional Animal Care and Use Committee of Weill Cornell Medicine. For the experimental metastasis assay, either 1 x 10^6^ N134 cells in 100 μL PBS were injected into the tail veins of NSG mice or 1 x 10^6^ BON1-TGL cells in 100 μL PBS were injected into the left ventricle of NSG mice. The orthotopic model of PanNET liver metastasis was performed as previously described [11] with the following modification: 0.5 x 10^6^ BON1-TGL cells in 50 μL DMEM containing 2% FBS were injected into the spleen. A standard formula for tumor volume was applied (volume [mm^3^] = 0.52 × width^2^ × length). Tumor burden is the sum of the tumor volume per mouse.

**References for Methods and Materials**

1. Evers BM, Townsend CM, Jr., Upp JR, Allen E, Hurlbut SC, Kim SW, Rajaraman S, Singh P, Reubi JC, Thompson JC: **Establishment and characterization of a human carcinoid in nude mice and effect of various agents on tumor growth.** *Gastroenterology* 1991, **101:**303-311.

2. Zhou Y, Park SY, Su J, Bailey K, Ottosson-Laakso E, Shcherbina L, Oskolkov N, Zhang E, Thevenin T, Fadista J, et al: **TCF7L2 is a master regulator of insulin production and processing.** *Hum Mol Genet* 2014, **23:**6419-6431.

3. Fadista J, Vikman P, Laakso EO, Mollet IG, Esguerra JL, Taneera J, Storm P, Osmark P, Ladenvall C, Prasad RB, et al: **Global genomic and transcriptomic analysis of human pancreatic islets reveals novel genes influencing glucose metabolism.** *Proc Natl Acad Sci U S A* 2014, **111:**13924-13929.

4. Taneera J, Fadista J, Ahlqvist E, Atac D, Ottosson-Laakso E, Wollheim CB, Groop L: **Identification of novel genes for glucose metabolism based upon expression pattern in human islets and effect on insulin secretion and glycemia.** *Hum Mol Genet* 2015, **24:**1945-1955.

5. Tang LH, Contractor T, Clausen R, Klimstra DS, Du YC, Allen PJ, Brennan MF, Levine AJ, Harris CR: **Attenuation of the retinoblastoma pathway in pancreatic neuroendocrine tumors due to increased cdk4/cdk6.** *Clinical cancer research : an official journal of the American Association for Cancer Research* 2012, **18:**4612-4620.

6. Du YC, Chou CK, Klimstra DS, Varmus H: **Receptor for hyaluronan-mediated motility isoform B promotes liver metastasis in a mouse model of multistep tumorigenesis and a tail vein assay for metastasis.** *Proceedings of the National Academy of Sciences of the United States of America* 2011, **108:**16753-16758.

7. Holland EC, Hively WP, DePinho RA, Varmus HE: **A constitutively active epidermal growth factor receptor cooperates with disruption of G1 cell-cycle arrest pathways to induce glioma-like lesions in mice.** *Genes Dev* 1998, **12:**3675-3685.

8. Du YC, Lewis BC, Hanahan D, Varmus H: **Assessing tumor progression factors by somatic gene transfer into a mouse model: Bcl-xL promotes islet tumor cell invasion.** *PLoS biology* 2007, **5:**e276.

9. Choi S, Chen Z, Tang LH, Fang Y, Shin SJ, Panarelli NC, Chen YT, Li Y, Jiang X, Du YC: **Bcl-xL promotes metastasis independent of its anti-apoptotic activity.** *Nat Commun* 2016, **7:**10384.

10. Zhang G, Chi Y, Du YN: **Identification and Characterization of Metastatic Factors by Gene Transfer into the Novel RIP-Tag; RIP-tva Murine Model.** *J Vis Exp* 2017.

11. Zhang G, Du YN: **Orthotopic Pancreatic Tumor Mouse Models of Liver Metastasis.** *Methods Mol Biol* 2019, **1882:**309-320.
